# The epidemiology of mild traumatic brain injury: the Trondheim MTBI follow-up study

**DOI:** 10.1186/s13049-018-0495-0

**Published:** 2018-04-27

**Authors:** Toril Skandsen, Cathrine Elisabeth Einarsen, Ingunn Normann, Stine Bjøralt, Rune Hatlestad Karlsen, David McDonagh, Tom Lund Nilsen, Andreas Nylenna Akslen, Asta Kristine Håberg, Anne Vik

**Affiliations:** 1 Department of Neuromedicine and Movement Science, Faculty of Medicine and Health Sciences, Norwegian University of Science and Technology, Trondheim, Norway; 2 Department of Physical Medicine and Rehabilitation, St. Olavs Hospital, Trondheim University Hospital, Trondheim, Norway; 3 Orthopaedic Department, St. Olavs Hospital, Trondheim University Hospital, Trondheim, Norway; 4 Municipal Emergency Department, Trondheim kommune, Trondheim, Norway; 5 Department of Radiology and Nuclear Medicine, St. Olavs Hospital, Trondheim University Hospital, Trondheim, Norway; 6 Department of Neurosurgery, St. Olavs Hospital, Trondheim University Hospital, Trondheim, Norway; 7 Department of Public Health and Nursing, NTNU, Faculty of Medicine and Health Sciences, Trondheim, Norway

**Keywords:** Cohort studies, Mild traumatic brain injury, CT, Medical care, Epidemiology

## Abstract

**Background:**

Mild traumatic brain injury (MTBI) is a frequent medical condition, and some patients report long-lasting problems after MTBI. In order to prevent MTBI, knowledge of the epidemiology is important and potential bias in studies should be explored.

Aims of this study were to describe the epidemiological characteristics of MTBI in a Norwegian area and to evaluate the representativeness of patients successfully enrolled in the Trondheim MTBI follow-up study.

**Methods:**

During 81 weeks in 2014 and 2015, all persons aged 16–60 years, presenting with possible MTBI to the emergency department (ED) at St. Olavs Hospital, Trondheim University Hospital or to Trondheim municipal outpatient ED, were evaluated for participation in the follow-up study. Patients were identified by CT referrals and patient lists. Patients who were excluded or missed for enrolment in the follow-up study were recorded.

**Results:**

We identified 732 patients with MTBI. Median age was 28 years, and fall was the most common cause of injury. Fifty-three percent of injuries occurred during the weekend. Only 29% of MTBI patients were hospitalised. Study specific exclusion criteria were present in 23%. We enrolled 379 in the Trondheim MTBI follow-up study. In this cohort, Glasgow Coma Scale score was 15 at presentation in 73%; 45% of patients were injured under the influence of alcohol. Patients missed for inclusion were significantly more often outpatients, females, injured during the weekend, and suffering violent injuries, but differences between enrolled and not enrolled patients were small.

**Conclusion:**

Two thirds of all patients with MTBI in the 16–60 age group were treated without hospital admission, patients were often young, and half of the patients presented during the weekend. Fall was the most common cause of injury, and patients were commonly injured under the influence of alcohol, which needs to be addressed when considering strategies for prevention. The Trondheim MTBI follow-up study comprised patients who were highly representative for the underlying epidemiology of MTBI.

## Background

In the past decade, there has been an enormous interest in the study of MTBI. This has been reflected by hundreds of published articles [1, 2], multi-site studies in Europe [3, 4] and the United States [5] and numerous consensus and agreement statements being published related to sport-related concussion [6, 7].

For the European Union, it was recently estimated that 2.5 mill cases of TBI occur every year [8], and since at least 90% of the cases of TBI are mild [9], evidently MTBI is a major health issue. The fact that a subgroup of the patients with MTBI report long-lasting symptoms interfering with their daily life [2], calls for better strategies for injury prevention. In a recent review, a key message was that TBI is, to great extent, preventable. It was further emphasised that strategies to lower the occurrence of injuries should be based on knowledge of the epidemiology, causes of TBI and risk groups, and that especially for MTBI, the monitoring of the epidemiology was incomplete [8].

In the existing literature, reports of the epidemiological characteristics and outcomes of MTBI are diverging. The differences may reflect true variations, but also differences in studies when it comes to setting, case ascertainment, recruitment procedures and exclusion criteria. Importantly, the large group of patients with uncomplicated MTBI who are treated outside hospitals, is likely to be underrepresented in studies [10].

In the Nordic countries, most existing epidemiological studies on MTBI were conducted decades ago, and mostly included only patients who were hospital-referred [11] or hospitalised for TBI [12–16]. Only a few studies included both hospitalised and non-hospitalised patients [17–19], but none of these dealt exclusively with MTBI. Hence, updated Nordic epidemiological studies of MTBI comprising both the hospital and the primary health care setting are needed.

Also within a given setting, study samples may not represent the heterogeneous group of patients presenting with MTBI. Notably, among MTBI prognosis studies reviewed, only 29% had low risk of bias [2]. Moreover, very broad exclusion criteria have been used, and some previous MTBI studies have actually excluded most of the available patients [20–22].

We therefore put a large effort into to enrolling as many patients with MTBI as possible, applying few exclusion criteria, into the Trondheim MTBI follow-up study in Norway; a study where we explore the pathophysiology and outcome of MTBI in a longitudinal design, applying also advanced MRI examinations, assessment of cognition and measurement of blood biomarkers. The cohort was established by prospectively identifying and reviewing all cases aged 16–60 years who presented with a possible MTBI to the municipal emergency department (ED) and the ED at a level 1 trauma centre.

The aim of the current study was to describe the epidemiological characteristics of all the evaluated patients with MTBI aged 16–60 in a Norwegian area. The second aim was to describe and examine the completeness and representativeness of the sample of patients eventually enrolled in the Trondheim MTBI follow-up study.

## Methods

### Study period, site, and population

In this prospective study, the inclusion of MTBI patients was from April 1st, 2014- to December 5th 2015. The inclusion was paused during vacation and holidays for a total of 7 weeks, resulting in 81 weeks of patient inclusion. Patients were recruited from two emergency departments (ED): St. Olav’s Hospital, Trondheim University Hospital, a Norwegian regional level 1 trauma centre and Trondheim Municipal Emergency clinic (only out-patients), also located at the hospital. The EDs use the same CT service. Their main catchment area for MTBI is the city of Trondheim and four neighbouring municipal entities with a total of 229,000 residents. In addition, Trondheim is a university city with around 18,000 students who come from other areas in Norway. The EDs, like most health care in Norway, are state run. During normal weekday work hours, patients can contact either their general practitioner (GP) or one of the EDs, while outside office working hours, patients present to the EDs.

### Inclusion and exclusion criteria in Trondheim MTBI follow-up study

Inclusion criteria were (1) having sustained MTBI and (2) age 16.0–59.9 years. The upper limit was chosen since participants were also invited to MRI, and the burden of non-traumatic abnormalities increases with age, making it more difficult to study the impact of the MTBI. We applied the recent definition of *TBI*, defining TBI as “an alteration in brain function, or other evidence of brain pathology, caused by an external force” [23]. In the present study, these criteria were operationalised as follows: we included patients who had experienced a physical trauma towards the head or high energy trauma, if either (1) witnessed loss of consciousness (LOC) or confusion and/or (2) self-reported amnesia for the event or the time period after the event, and/or (3) a traumatic brain lesions on CT was reported. Next, cases identified with *TBI* were further categorised as *mild* (MTBI) if they met the WHO criteria for being mild: Glasgow Coma Scale (GCS) score 13–15 at presentation, LOC < 30 min, and posttraumatic amnesia (PTA) < 24 h [1]. Evaluation of intoxicated patients represented a challenge, and we sought to be confident that their self-reported amnesia was a result of MTBI and not intoxication. Therefore, only patients who had been observed by the people accompanying them as fully conscious prior to the injury, or who reported complete memory for events immediately prior to the injury, were considered to have a MTBI.

MTBI was the chosen term in this study, acknowledging that the term “concussion” may be more common in the sports medicine literature [7]. Exclusion criteria in the Trondheim MTBI follow-up study were late presentation or presence of co-morbidities or circumstances that would make it very difficult to follow-up patients or where a valid MTBI-related outcome could not be obtained: (a) non-residency in Norway or non-fluency in the Norwegian language, (b) ongoing, severe psychiatric disease, severe somatic disease or drug abuse; that would complicate follow-up, (c) history of complicated mild, moderate or severe TBI or other neurological conditions with brain pathology visible on imaging or known cognitive deficits, (d) presentation > 48 h after the initial trauma, and (e) other concurrent major trauma, such as spinal cord injury, severe fractures or internal injuries.

### Study procedures

Recruiters and their supervisors screened all referrals to head CT and patient lists at the municipal ED 1–2 times daily and contacted the neurosurgical residents on call. If needed, they evaluated the potential participant’s medical record, or a study specific form, for inclusion and exclusion criteria. A log of the selection process was kept for CT referrals. Study personnel were present at the hospital for 8–12 h daily Monday to Friday, called in as required on Saturday and 5 h on Sunday.

Patients with a likely or possible MTBI were approached in the hospital ward or in the ED. Patients who had left the ED, were contacted by phone or text message. Subsequently, study personnel interviewed patients and evaluated their eligibility for the prospective cohort study, and information was sent via e-mail for consent. When attempt to contact failed, we used medical records information regarding GCS score, amnesia or LOC for MTBI criteria evaluation and inclusion eligibility.

Recruiters were PhD students and medical students. To ensure compliance with study protocol, all recruiters underwent training, participated in Good Clinical Practice courses and had access to supervision by consultants during their shifts.

Patients who had been referred directly to head CT from the general practitioners in the catchment area for MTBI were not evaluated for enrolment in the Trondheim MTBI follow-up study. As part of assessing the representativeness of the follow-up study, a medical student (IN) retrospectively evaluated all such CT referrals with regard to the study criteria.

### Study variables

Information came from patient interviews and medical records. The GCS score was recorded from the medical record or observed by the study personnel. If lacking, the history and clinical descriptions in the medical record were used to estimate a score. LOC was determined as present if witnessed. Duration of PTA was recorded as the time after injury for which the patient had no continuous memory (< 1 h, or 1–24 h). Head CT findings were obtained from the radiology report. Findings were categorised into: traumatic intracranial finding (with or without additional fractures), cranial fracture (without intracranial traumatic findings, with or without facial fractures) and facial fracture (without intracranial traumatic findings or cranial fracture). Self-reported time of injury or, if unknown, time of CT was used to determine time of injury. The weekend was defined as the time interval between Friday 16:00 to Sunday 24:00 and night as 22:00–07:00. Influence of alcohol was based on clinical description and self-report, and dichotomised into yes or no. Presence of other concurrent injuries was also dichotomised into as yes or no, based on self-report and records. Abrasions and contusions in the skin were not included.

### Statistical analyses

Descriptive statistics of the main study variables are presented as percentages or as median with interquartile range (IQR). We used Mann-Whitney U test to compare the median age of patients enrolled and not enrolled in the Trondheim MTBI follow-up study, whereas Chi-square tests were used to analyse categorical variables. We also used logistic regression to calculate odds ratio (OR) for dichotomous variable between patients enrolled and not enrolled in the Trondheim MTBI follow-up study. We used IBM© Statistical Package for the Social Sciences (SPSS©) Statistics version 22.

## Results

### Patients identified with MTBI during the study period

**Fig. 1:**
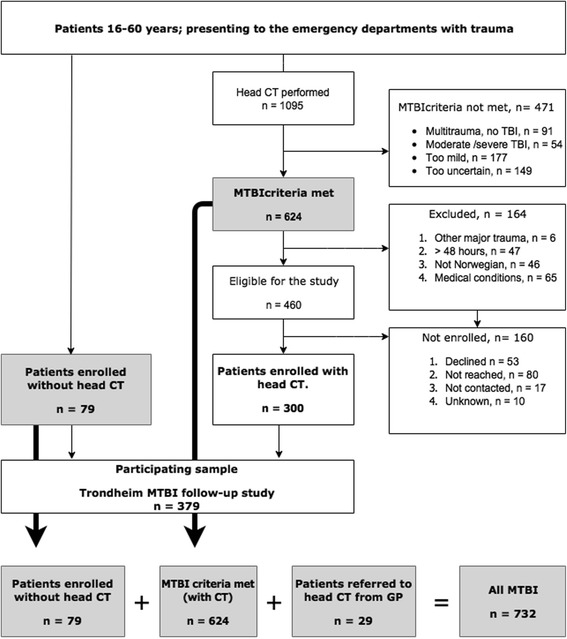
Flow chart of the identification of patients and the enrolment into the Trondheim MTBI follow-up study. Legend: The filled boxes and the broad arrows shows the identification of all patients with MTBI in the study period. The boxes connected with thin narrows represent the recruitment to the Trondheim MTBI follow-up study. MTBI = mild traumatic brain injury; GP = general practitioner

During the study period, 1095 patients were examined with head CT due to trauma, 624 of these had a likely MTBI (Fig. 1). Furthermore, 79 patients who had not been examined with head CT were identified by screening of patients lists and subsequently enrolled in the Trondheim MTBI follow-up study. In addition, we retrospectively identified 29 patients who had been referred directly to CT from a GP. Hence, a total of 732 patients were identified with MTBI (Fig. 1).

### Patients enrolled into the Trondheim MTBI follow-up study

Out of the 624 patients with MTBI who presented to the study EDs and had a head CT, 164 (26%) met one of the exclusion criteria, leaving 460 evaluated as eligible for the Trondheim MTBI follow-up study. Out of these, 300 were enrolled, which constituted 65% of the eligible patients and 48% of all patients with MTBI who presented to the study EDs and had a head CT. Only 53 declined participation, while 97 were not successfully approached. Together with the 79 patients identified and enrolled without head CT, in total, 379 patients were enrolled in the Trondheim MTBI follow-up study (Fig. 1).

### Epidemiological characteristics of all MTBI

The median age in the total sample was 28 years, and 62% were male. (Table 1). Half of the injuries (53%) occurred during the weekends, and 71% were treated without hospital admittance. Falls were the most common cause of injury, followed by violence, bicycle, sports and motor vehicle accidents (MVA). MVA accounted for only 11% of the cases. Fall and bicycle accidents were more common with increasing age, while violence, sports and MVA was more common among the youngest (Table 2). Of all patients with MTBI examined with head CT, 6% had intracranial traumatic findings (Table 1).

**Table 1:** Patient characteristics in all patients with mild traumatic brain injury and in the cohort Trondheim MTBI follow-up study

Variable	All patients identified with MTBI	Trondheim MTBI follow-up study
	*n* = 732	*n* = 379
Age, median [IQR]	28 [21,42]	25 [20,40]
Age, mean (SD)	31.7 (12.7)	31.2 (13.0)
Male, n (%)	450 (62)	247 (65)
Cause of injury		
Fall, n (%)	267 (37)	136 (36)
Violence, n (%)	144 (20)	65 (17)
Bicycle, n (%)	98 (13)	58 (15)
Sport, n (%)	81 (11)	54 (14)
MVA, n (%)	83 (11)	43 (11)
Struck object, n (%)	37 (5)	17 (5)
Other, n (%)	9 (1)	3 (1)
Unknown, n (%)	13 (2)	3 (1)
Alcohol influence, n (%)	unknown	169 (45)
Time of injury		
Weekend, n (%)	38 (53)^a^	210 (55)
Head CT performed (%)		300 (79)
Intracranial traumatic CT findings (yes/no)	37 (6)	21 (7)
GCS score, n (%)		
15	495 (67)	278 (73)
14	86 (12)	57 (15)
13	9 (1)	5 (1)
Missing^b^	142 (19)	39 (10)
LOC, yes (%)	unknown	175 (46)
PTA	unknown	379 (100)
< 1 h (%)		272 (72)
1–24 h (%)		107 (28)
Other injuries, yes (%)	unknown	133 (35)
Level of care, n (%)		
Discharged home from ED	517 (71)	261 (69)
Observation < 24 h	89 (12)	61 (16)
Neurosurgical admission	69 (9)	39 (10)
Another admission	50 (7)	18 (5)
Missing	7 (1)	–

*Abbreviations*: *MTBI* mild traumatic brain injury, *MVA* motor vehicle accidents, *GCS* Glasgow coma scale, *LOC* loss of consciousness, *PTA* posttraumatic amnesia, *ED* emergency department

^a^Time of CT

^b^In 17 out of the 39 patients with missing GCS score, the score could reliably be evaluated to either 14 or 15 based on the medical record

**Table 2:** Sex distribution and causes of mild traumatic brain injury by age in all 732 cases

	Age groups (years)									
	All	16–20	21–25	26–30	31–35	36–40	41–45	46–50	51–55	56–60
	*N* = 732	*n* = 175	*n* = 153	*n* = 87	*n* = 68	n = 46	*n* = 42	*n* = 72	*n* = 54	*n* = 35
Sex, male	62%	55%	63%	62%	65%	72%	62%	67%	59%	60%
Cause of injury n (%)										
Fall	267 (37)	51 (29)	50 (33)	29 (33)	24 (35)	15 (33)	15 (36)	34 (47)	27 (50)	22 (63)
Violence	144 (20)	39 (22)	39 (26)	21 (24)	13 (19)	11 (24)	4 (10)	9 (13)	5 (9)	3 (9)
Bicycle	98 (13)	11 (6)	14 (9)	8 (9)	12 (18)	9 (20)	7 (17)	17 (24)	11 (20)	9 (26)
Sports	81 (11)	33 (19)	25 (16)	5 (6)	4 (6)	5 (11)	5 (12)	3 (4)	1 (2)	–
MVA	83 (11)	32 (18)	9 (6)	14 (16)	6 (9)	4 (9)	5 (12)	6 (8)	6 (11)	1 (3)
Struck object	37 (5)	7 (4)	9 (6)	7 (8)	6 (9)	2 (4)	3 (7)	2 (3)	1 (2)	–
Other	9 (1)	1 (1)	3 (2)	1 (1)	2 (3)	–	–	1 (1)	1 (2)	–
Unknown	13 (2)	1 (1)	4 (3)	2 (2)	1 (2)	–	3 (7)	–	2 (4)	–

*Abbreviation*: *MVA* motor vehicle accidents

### Additional characteristics of MTBI in the Trondheim MTBI follow-up study

In the Trondheim MTBI follow-up study sample, more detailed clinical information was available. In total, 45% were injured under the influence of alcohol; 92% of patients injured due to violence, 57% of patients who had fallen and 14% of patients injured in MVA were injured while influenced by alcohol. Median age in the intoxicated patients was 24.5 years, 72% were male, 76% of the intoxicated patient presented in the weekend and 88% at night.

GCS score at presentation was 15 in 73% of the cases, and only 1% had GCS score 13. LOC was observed in 46% of the cases, with a LOC < 5 min in 89% of patients with LOC. PTA was reported by all and lasted < 1 h in 72%. Other injuries were registered in 35% of patients included in Trondheim MTBI follow-up study; mainly muscular-skeletal complaints, facial injuries and wounds needing suturing (Table 1).

The patients who had not been examined with CT, were more likely to be discharged home from the ED (*p* < 0.001), were less likely to have a GCS score of < 15 (*p* = 0.007), and more likely to have a PTA duration < 1 h (81% vs 68%, *p* = 0.027) compared to patients examined with CT.

### Patients with MTBI who met the exclusion criteria for the Trondheim MTBI follow-up study

Compared to the enrolled patients (with CT) (*n* = 300), median age was higher in patients who met one of the exclusion criteria for the Trondheim MTBI follow-up study (*n* = 164); 34 years, (IQR; 26–47), *p* < 0.001. The reasons for exclusion were medical conditions (including substance abuse) (*n* = 65; 40%), presenting late (*n* = 47; 29%), not being Norwegian (*n* = 46; 28%) and having other major trauma (*n* = 6; 4%) (Fig. 1).

### Eligible patients who were not enrolled in the Trondheim MTBI follow-up study

The 460 patients examined with head CT and meeting the inclusion criteria for the follow-up study; i.e. who were eligible patients (Fig. 1), were further analysed to examine differences in patient characteristics between patients enrolled and not enrolled in the Trondheim MTBI follow-up study (Table 3). The 160 eligible patients who were not enrolled, were more likely to be females or to have sustained injury by violence, were more often injured during the weekend, and were more often discharged home from the ED. The differences between enrolled and not enrolled patients were small (see odds ratios, Table 3).

**Table 3:** Characteristics of patients enrolled and not enrolled in the Trondheim MTBI follow-up study based on head CT referrals

Variable	Enrolled	Not enrolled	*p*-value	OR^a^ (95% CI)
	*n* = 300	*n* = 160		
Age, Median [IQR]	25 [20,43]	25 [20,38]	0.12	
Age, mean (SD)	31.5 (13.3)	29.4 (11.9)	NC	
Male, n (%)	195 (65)	88 (55)	0.036	0.66 (0.45–0.97)
Injury cause, n (%)				
Fall	105 (35)	58 (36)	0.79	1.06 (0.71–1.58)
Violence	47 (16)	41 (26)	0.01	1.86 (1.16–2.97)
Bicycle	52 (17)	17 (11)	0.06	0.58 (0.32–1.04)
Sport	45 (15)	16 (10)	0.13	0.63 (0.34–1.15)
Motor vehicle accident	36 (12)	18 (11)	0.81	0.93 (0.51–1.70)
Struck object	10 (3)	3 (2)	0.48	0.62 (0.17–2.32)
Other or unknown	5 (2)	7 (4)	0.95	2.7 (0.84–8.65)
Time of CT, n (%)				
During weekend, Yes (%)	163 (54)	104 (65)	0.027	1.56 (1.05–2-32)
During night, Yes %	130 (43)	75 (47)	0.47	1.15 (0.79–1.70)
GCS score, n (%)				
GCS score 15^b^	216 (79)	81 (82)	0.53	1.21 (0.67–2.17)
CT findings, n (%)				
Intracranial lesions	21 (7)	7 (4)	0.27	0.61^b^ (0.25–1.46)
Cranial fractures	4 (1)	0		NC
Facial fractures	18 (6)	8 (5)		NC
Level of care, n (%)				
Discharged home from ED	184 (61)	122 (76)	0.001	0.49^b^ (0.32–0.76)

*Abbreviations*: *CI* confidence interval, *NC* not calculated, *GCS* Glasgow coma scale, *OR* odds ratio for not being enrolled

^a^binary logistic regression with this category coded 0 and all other coded 1

^b^87 cases without valid GCS score not included in the analysis

### Patients referred directly to head CT by general practitioners

Twenty-nine patients with MTBI were referred directly to CT by their GP. Out of these, 16 would most likely have met the inclusion criteria (eligible) for the follow-up study while 13 would have been excluded (presentation > 48 h after the injury). In the patients who would have been eligible, median age was 30 years (IQR; 23–41), 56% were female and they were frequently injured by hitting an object (31%). The head CT was normal in all and none was admitted to hospital.

## Discussion

To identify cases of MTBI, we evaluated all cases of head injuries in persons between 16 and 60 years presenting to either the regional trauma centre, the municipal out-patient emergency clinic or the GPs in the catchment area. More than two thirds of patients with MTBI were treated without hospital admittance, and only 6% had traumatic intracranial findings on head CT. Almost half of the enrolled patients were injured under the influence of alcohol. We managed to enrol around half of all patients with MTBI, into the Trondheim MTBI follow-up study. Notably, the differences in demographic and clinical characteristics between enrolled and not enrolled eligible patients were small.

### Epidemiological characteristics of MTBI

In the present study, in line with the studies of Styrke et al. [18] and Ingebrigtsen et al. [19], MTBI was especially common in the young. In adolescents, falls, violence, sports activities and MVA were all common causes, indicating that a more active, and possibly more carefree, lifestyle involves a higher risk of trauma.

Only 11% were injured in MVA, both in the total material and in the Trondheim MTBI follow-up study. The low proportion of MVA was is in accordance with results from studies of isolated TBI [24], and studies of patients not admitted to hospital [25], but lower than several mixed samples [26, 27]. We consider that the low proportion of MVA reflects that many MTBIs result from more trivial accidents, and that these were clearly captured in this study. It should, however, be noted that Norway has a low incidence of traffic related injuries in general. Notably, Norway was recently recognised by the EU for having the highest reduction in road traffic deaths among 32 monitored European countries (http://etsc.eu/10th-annual-road-safety-performance-index-pin-report/). The low proportion of MVA in the mild TBI cohort could therefore partly reflect the high road traffic security in Norway.

### Clinical characteristics of MTBI

An interesting finding was that only 1% of MTBI patients presented with a GCS score of 13. The reason for this low percentage is probably that few patients with GCS 13 report PTA < 24 h when evaluated carefully. The low frequency of GCS score of 13 in MTBI lends validity to a definition of TBI as moderate when GCS score is 9–13, as applied in several studies and classifications [28, 29].

Further, less than one third of the MTBI cases were admitted, both in the total material and in the Trondheim MTBI follow-up study, to hospital. Hence, we succeeded in recruiting patients with the expected epidemiology of MTBI [18, 30–32].

As expected, intracranial traumatic CT findings were uncommon in MTBI, but were seen also in patients who presented with a GCS score of 15.

The percentage of patients influenced by alcohol was high, 45%, even higher than reported in earlier [18] or more recent studies of MTBI [33]. In a Norwegian study of trauma patients, 28% of the injuries were alcohol-related [34]. The high frequency in the current study might partly reflect the large number of students living in the catchment area.

Alcohol use is a significant risk factor for injuries [35, 36] and the risk increases with the frequency of heavy episodic drinking [37], a pattern of alcohol use found to be more common in the Nordic countries than in the south of Europe [34]. A striking finding in our study, was that almost all of the patients who were injured by violence, were influenced by alcohol. The relationship between alcohol consumption and violent behaviour is well known, and again, the prevalence of alcohol-related aggression has been found to be higher in cultures where intoxication is common, like in Norway [34]. Not surprisingly, most of the patients who were influenced by alcohol presented in the weekends and nights, which demonstrates the challenges with recruitment of patients with MTBI in research studies.

### Representativeness of the Trondheim MTBI follow-up study

We managed to enrol 65% of eligible patients into the Trondheim MTBI follow-up study, which was higher than reported in previous studies [20–22]. Nevertheless, our results suggest that patients were not missed at random. Indeed, more of the patients injured during the weekend, by violence, and non-hospitalised were missed for enrolment. However, the differences in demographic and clinical characteristics between the enrolled patients and those missed for enrolment were small.

A surprising finding was that one quarter of the patients with MTBI met the exclusion criteria in the follow-up study even though the exclusion criteria were solely designed to prevent enrolment of patients considered impossible to follow up, or whose outcome would not be valid. That such a large number of potential participants had to be excluded, demonstrates that results from follow-up studies in patients with MTBI, inevitably will suffer from some degree of selection bias. Patients with ongoing substance abuse for example, are susceptible to fall and sustain a TBI, while their clinical course and outcomes remains poorly studied. Still, the rate of exclusion was lower than in previous studies. In a Finnish study of MTBI in healthy adults of working age, many patients were not enrolled due to pre-existing health conditions or other exclusion criteria, leading to a low percentage of enrolment of patients screened for inclusion [21]. Trondheim MTBI follow-up study addresses the issue with high rates of exclusion, by a broader inclusion. That the differences between enrolled and not enrolled patients in the present study in general were small, is promising regarding the generalisability of the future results in the Trondheim MTBI follow-up study.

### Strengths and limitations

The main strengths and novelty of this study were that we also included patients who were evaluated in the primary care setting, and that all medical records and CT referrals were prospectively reviewed for case ascertainment. Limitation were that we only studied patients 16 and 60 years of age. Moreover, regarding the patients who were not referred to CT, epidemiological data were collected only for those enrolled.

## Conclusion

We prospectively evaluated all MTBI in a catchment area capturing both hospital admittances and outpatient municipal ED and GP visits. The Trondheim MTBI follow-up study comprised around half of all the patients presenting with MTBI, and we could demonstrate that the cohort was largely representative of all the patients seen with MTBI. Nevertheless, this study demonstrates the challenge with recruitment of patients with MTBI, typically young patients with uncomplicated MTBI, who tend to present during weekends and at night-time. Moreover, the results of this study indicate that prevention of MTBI is a big challenge. To reduce the occurrence of MTBI it will be necessary to address the culture of high and frequent consumption of alcohol in our society and the life style of young people. Finally, the effect of MTBI in persons with severe medical conditions, like on-going substance abuse, still needs to be studied.

## Abbreviations

CI: Confidence interval
ED: Emergency department
GCS: Glasgow Coma Scale
GP: General practitioner
IQR: Interquartile range
LOC: Loss of consciousness
MTBI: Mild traumatic brain injury
MVAs: Motor vehicle accidents
OR: Odds ratio
PTA: Posttraumatic amnesia

## References

[1] Carroll LJ, Cassidy JD, Holm L, Kraus J, Coronado VG. Methodological issues and research recommendations for mild traumatic brain injury: the WHO Collaborating Centre Task Force on mild traumatic brain injury. J Rehabil Med. 2004;36(43 Suppl):113–25. 10.1080/16501960410023877 15083875

[2] Cassidy JD, Cancelliere C, Carroll LJ, Cote P, Hincapie CA, Holm LW, Hartvigsen J, Donovan J, Nygren-de Boussard C, Kristman VL (2014). Systematic review of self-reported prognosis in adults after mild traumatic brain injury: results of the International Collaboration on mild traumatic brain injury prognosis. Arch Phys Med Rehabil.

[3] Maas AI, Menon DK, Steyerberg EW, Citerio G, Lecky F, Manley GT, Hill S, Legrand V, Sorgner A, Participants C-T (2015). Collaborative European NeuroTrauma Effectiveness Research in Traumatic Brain Injury (CENTER-TBI): a prospective longitudinal observational study. Neurosurgery.

[4] Foks KA, Cnossen MC, Dippel DWJ, Maas A, Menon D, van der Naalt J, Steyerberg EW, Lingsma H, Polinder S. Management of mild traumatic brain injury at the emergency department and hospital admission in Europe: a survey of 71 neurotrauma centers participating in the CENTER-TBI study. J Neurotrauma. 2017; 10.1089/neu.2016.4919. 10.1089/neu.2016.4919 28398105

[5] Yue JK, Vassar MJ, Lingsma HF, Cooper SR, Okonkwo DO, Valadka AB, Gordon WA, Maas AI, Mukherjee P, Yuh EL (2013). Transforming research and clinical knowledge in traumatic brain injury pilot: multicenter implementation of the common data elements for traumatic brain injury. J Neurotrauma.

[6] Giza CC, Kutcher JS, Ashwal S, Barth J, Getchius TS, Gioia GA, Gronseth GS, Guskiewicz K, Mandel S, Manley G (2013). Summary of evidence-based guideline update: evaluation and management of concussion in sports: report of the Guideline Development Subcommittee of the American Academy of Neurology. Neurology.

[7] McCrory P, Meeuwisse W, Dvorak J, Aubry M, Bailes J, Broglio S, Cantu RC, Cassidy D, Echemendia RJ, Castellani RJ (2017). Consensus statement on concussion in sport-the 5th international conference on concussion in sport held in Berlin, October 2016. Br J Sports Med.

[8] Maas AIR, Menon DK, Adelson PD, Andelic N, Bell MJ, Belli A, Bragge P, Brazinova A, Buki A, Chesnut RM (2017). Traumatic brain injury: integrated approaches to improve prevention, clinical care, and research. Lancet Neurol.

[9] Feigin VL, Theadom A, Barker-Collo S, Starkey NJ, McPherson K, Kahan M, Dowell A, Brown P, Parag V, Kydd R (2013). Incidence of traumatic brain injury in New Zealand: a population-based study. Lancet Neurol.

[10] Corrigan JD, Selassie AW, Orman JA (2010). The epidemiology of traumatic brain injury. J Head Trauma Rehabil.

[11] Heskestad B, Baardsen R, Helseth E, Romner B, Waterloo K, Ingebrigtsen T (2009). Incidence of hospital referred head injuries in Norway: a population based survey from the Stavanger region. Scand J Trauma Resusc Emerg Med.

[12] Pedersen K, Fahlstedt M, Jacobsson A, Kleiven S, von Holst H (2015). A national survey of traumatic brain injuries admitted to hospitals in Sweden from 1987 to 2010. Neuroepidemiology.

[13] Engberg AW, Teasdale TW (2004). A population-based study of survival and discharge status for survivors after head injury. Acta Neurol Scand.

[14] Andelic N, Sigurdardottir S, Brunborg C, Roe C (2008). Incidence of hospital-treated traumatic brain injury in the Oslo population. Neuroepidemiology.

[15] Edna TH, Cappelen J (1984). Hospital admitted head injury. A prospective study in Trondelag, Norway, 1979-80. Scand J Soc Med.

[16] Peloso PM, von Holst H, Borg J, et al. J Rehabil Med. 2004;36(43 Suppl):22–7. 10.1080/16501960410023714 15083869

[17] Andersson EH, Bjorklund R, Emanuelson I, Stalhammar D (2003). Epidemiology of traumatic brain injury: a population based study in western Sweden. Acta Neurol Scand.

[18] Styrke J, Stalnacke BM, Sojka P, Bjornstig U (2007). Traumatic brain injuries in a well-defined population: epidemiological aspects and severity. J Neurotrauma.

[19] Ingebrigtsen T, Mortensen K, Romner B (1998). The epidemiology of hospital-referred head injury in northern Norway. Neuroepidemiology.

[20] Luoto TM, Tenovuo O, Kataja A, Brander A, Ohman J, Iverson GL (2013). Who gets recruited in mild traumatic brain injury research?. J Neurotrauma.

[21] Isokuortti H, Iverson GL, Kataja A, Brander A, Ohman J, Luoto TM (2016). Who gets head trauma or recruited in mild traumatic brain injury research?. J Neurotrauma.

[22] McCullagh S, Feinstein A (2003). Outcome after mild traumatic brain injury: an examination of recruitment bias. J Neurol Neurosurg Psychiatry.

[23] Menon DK, Schwab K, Wright DW, Maas AI (2010). Position statement: definition of traumatic brain injury. Arch Phys Med Rehabil.

[24] Ratcliff JJ, Adeoye O, Lindsell CJ, Hart KW, Pancioli A, McMullan JT, Yue JK, Nishijima DK, Gordon WA, Valadka AB (2014). ED disposition of the Glasgow coma scale 13 to 15 traumatic brain injury patient: analysis of the transforming research and clinical knowledge in TBI study. Am J Emerg Med.

[25] de Koning ME, Scheenen ME, van der Horn HJ, Hageman G, Roks G, Spikman JM, van der Naalt J (2017). Non-hospitalized patients with mild traumatic brain injury: the forgotten minority. J Neurotrauma.

[26] de Koning ME, Scheenen ME, van der Horn HJ, Hageman G, Roks G, Yilmaz T, Spikman JM, van der Naalt J. Outpatient follow-up after mild traumatic brain injury: results of the UPFRONT-study. Brain Inj. 2017;31(8):1102–8. 10.1080/02699052.2017.1296193 28481634

[27] McMahon P, Hricik A, Yue JK, Puccio AM, Inoue T, Lingsma HF, Beers SR, Gordon WA, Valadka AB, Manley GT (2014). Symptomatology and functional outcome in mild traumatic brain injury: results from the prospective TRACK-TBI study. J Neurotrauma.

[28] Stein SC, Spettell C (1995). The Head Injury Severity Scale (HISS): a practical classification of closed-head injury. Brain Inj.

[29] Fabbri A, Servadei F, Marchesini G, Stein SC, Vandelli A (2008). Early predictors of unfavourable outcome in subjects with moderate head injury in the emergency department. J Neurol Neurosurg Psychiatry.

[30] Colantonio A, Saverino C, Zagorski B, Swaine B, Lewko J, Jaglal S, Vernich L (2010). Hospitalizations and emergency department visits for TBI in Ontario. Can J Neurol Sci.

[31] Korley FK, Kelen GD, Jones CM, Diaz-Arrastia R. Emergency department evaluation of traumatic brain injury in the United States, 2009-2010. J Head Trauma Rehabil. 2016;31(6):379–87. 10.1097/HTR.0000000000000187 PMC4786477 26360006

[32] Kerr ZY, Harmon KJ, Marshall SW, Proescholdbell SK, Waller AE (2014). The epidemiology of traumatic brain injuries treated in emergency departments in North Carolina, 2010-2011. N C Med J.

[33] Scheenen ME, de Koning ME, van der Horn HJ, Roks G, Yilmaz T, van der Naalt J, Spikman JM (2016). Acute alcohol intoxication in patients with mild traumatic brain injury: characteristics, recovery, and outcome. J Neurotrauma.

[34] Bye EK, Rossow I (2010). The impact of drinking pattern on alcohol-related violence among adolescents: an international comparative analysis. Drug Alcohol Rev.

[35] Borges G, Cherpitel C, Orozco R, Bond J, Ye Y, Macdonald S, Rehm J, Poznyak V (2006). Multicentre study of acute alcohol use and non-fatal injuries: data from the WHO collaborative study on alcohol and injuries. Bull World Health Organ.

[36] Borges G, Cherpitel CJ, Orozco R, Bond J, Ye Y, Macdonald S, Giesbrecht N, Stockwell T, Cremonte M, Moskalewicz J (2006). Acute alcohol use and the risk of non-fatal injury in sixteen countries. Addiction.

[37] Rossow I, Bogstrand ST, Ekeberg O, Normann PT (2013). Associations between heavy episodic drinking and alcohol related injuries: a case control study. BMC Public Health.

